# Treatment of COVID-19: old tricks for new challenges

**DOI:** 10.1186/s13054-020-2818-6

**Published:** 2020-03-16

**Authors:** Anne Catherine Cunningham, Hui Poh Goh, David Koh

**Affiliations:** 1 Pengiran Anak Puteri Rashidah Sa’adatul Bolkiah (PAPRSB), Institute of Health Sciences, Universiti Brunei Darussalam, Jalan Tungku Link, Gadong, BE1410 Brunei; 2 Saw Swee Hock School of Public Health, National University of Singapore, Singapore, Singapore

## Challenges in treating COVID-19

Coronavirus disease (COVID-19), which appeared in December 2019, presents a global challenge, particularly in the rapid increase of critically ill patients with pneumonia and absence of definitive treatment. To date, over 81,000 cases have been confirmed, with over 2700 deaths. The mortality appears to be around 2%; early published data indicate 25.9% with SARS-CoV-2 pneumonia required ICU admission and 20.1% developed acute respiratory distress syndrome [1].

There is presently no vaccine or specific anti-viral drug regime used to treat critically ill patients. The management of patients mainly focuses on the provision of supportive care, e.g., oxygenation, ventilation, and fluid management. Combination treatment of low-dose systematic corticosteroids and anti-virals and atomization inhalation of interferon have been encouraged as part of critical COVID-19 management [2]. Other reported therapeutic agents that are used for the treatment of seriously ill patients have been noted in Table 1.

**Table 1:** Potential treatment options of COVID-19

Classes	Potential treatment options	Reference
Anti-viral	> 85% of patients received anti-viral agents, including oseltamivir (75 mg every 12 h orally), ganciclovir (0.25 g every 12 h intravenously), and lopinavir/ritonavir tablets (400/100 mg twice daily). Remdesivir is currently under trials at more than ten medical institutions in Wuhan and has been known to prevent MERS-CoV.	[1]
Anti-malarial	An old anti-malarial, chloroquine phosphate, has been effective in inhibiting the exacerbation of pneumonia due to its anti-viral and anti-inflammatory activities.	[3]
Herbal treatments	There was widespread use of Traditional Chinese Medicine during the last SARS-COV outbreak and it is currently being used in China. The five most commonly used herbs were *Astragali Radix* (Huangqi), *Glycyrrhizae Radix Et Rhizoma* (Gancao), *Saposhnikoviae Radix* (Fangfeng), *Atractylodis Macrocephalae Rhizoma* (Baizhu), and *Lonicerae Japonicae Flo.*	[4]

## Convalescent plasma: one of the forgotten immunologically based strategies

Passive immunization has been successfully used to treat infectious diseases. A meta-analysis demonstrated a significant reduction in mortality and viral load in studies using convalescent plasma for the treatment of severe acute viral respiratory infections, including those caused by related coronaviruses (SARS-CoV and MERS-CoV) [5]. Serious adverse events were not reported. Eighty SARS patients were treated with convalescent plasma during the last major outbreak. A significantly better outcome was obtained with earlier transfusion (before day 14), and no immediate adverse events were observed.

A feasibility intervention study of convalescent plasma for MERS-CoV infection treatment failed to identify sufficient high-titer plasma from patients with confirmed/suspected MERS, their close family members, or healthcare workers exposed to MERS (*n* = 12 reactive ELISA/443 serum tested). Two fresh-frozen plasma units (250–350 mL/unit) would be required for each enrolled MERS patient (NCT02190799). Encouragingly, anti-MERS CoV titers measured by ELISA correlated with microneutralization (MN) assays [6].

There are currently over 30,000 recovered COVID-19 patients, who could present a valuable resource of convalescent plasma for health care providers. China National Biotec Group Co has claimed that 10 seriously ill patients receiving this immunoglobulin therapy demonstrated improved oxygenation and reduced inflammation and viral load [7]. High-titer specific antibodies should be able to bind to SARS-CoV-2 and neutralize the viral particles, block access to uninfected cells, and activate potent effector mechanisms (e.g., complement activation and phagocytosis).

## Potential risks and ethical considerations

It should be noted that treatment with human immunoglobulin has been associated with significantly increased same-day thrombotic event risk (0.04 to 14.9%) [8]. These data indicate the potential value of evaluating the effectiveness of early intervention therapy with convalescent plasma or SARS-CoV-2-specific hyperimmune globulin in patients with acute respiratory disease in this outbreak. Given the lack of knowledge on the basic biology of SARS-CoV-2, including virus variability and mutation, plasma collected locally may better reflect the circulating virus in the population and could be a valid treatment option.

Other issues to be considered include the lack of high-quality studies and the need for adequate selection of donors with high neutralizing antibody titers. It is also important to ensure that the production and the use of convalescent plasma take place according to precise ethical and controlled conditions for a possible role of these products of human origin [9].

## Conclusion

In the absence of definitive management protocols, many treatment regimes have been explored in the treatment of COVID-19. Some of these treatments may have been tried out of desperation, and among these, some show initial promise. However, it is too early to see any published results of rigorous clinical trials.

Using the serum of recovered patients is a tried and tested approach, and trials are underway to study its effectiveness. This treatment appears to be helpful in the short term until definitive and effective treatments are found.

## Data Availability

Not applicable
